# Large‐scale energy budget of impulsive magnetic reconnection: Theory and simulation

**DOI:** 10.1002/2016JA023169

**Published:** 2017-03-16

**Authors:** S. A. Kiehas, N. N. Volkonskaya, V. S. Semenov, N. V. Erkaev, I. V. Kubyshkin, I. V. Zaitsev

**Affiliations:** ^1^ Space Research InstituteAustrian Academy of SciencesGrazAustria; ^2^ Institute of PhysicsSt. Petersburg State UniversitySt. PetersburgRussia; ^3^ Institute of Computational ModellingRussian Academy of Sciences, Siberian BranchKrasnoyarskRussia; ^4^ Department of Computational PhysicsSiberian Federal UniversityKrasnoyarskRussia

**Keywords:** magnetic reconnection, plasma physics, magnetospheric physics, substorms

## Abstract

We evaluate the large‐scale energy budget of magnetic reconnection utilizing an analytical time‐dependent impulsive reconnection model and a numerical 2‐D MHD simulation. With the generalization to compressible plasma, we can investigate changes in the thermal, kinetic, and magnetic energies. We study these changes in three different regions: (a) the region defined by the outflowing plasma (outflow region, OR), (b) the region of compressed magnetic fields above/below the OR (traveling compression region, TCR), and (c) the region trailing the OR and TCR (wake). For incompressible plasma, we find that the decrease inside the OR is compensated by the increase in kinetic energy. However, for the general compressible case, the decrease in magnetic energy inside the OR is not sufficient to explain the increase in thermal and kinetic energy. Hence, energy from other regions needs to be considered. We find that the decrease in thermal and magnetic energy in the wake, together with the decrease in magnetic energy inside the OR, is sufficient to feed the increase in kinetic and thermal energies in the OR and the increase in magnetic and thermal energies inside the TCR. That way, the energy budget is balanced, but consequently, not all magnetic energy is converted into kinetic and thermal energies of the OR. Instead, a certain fraction gets transfered into the TCR. As an upper limit of the efficiency of reconnection (magnetic energy → kinetic energy) we find *η*_eff_=1/2. A numerical simulation is used to include a finite thickness of the current sheet, which shows the importance of the pressure gradient inside the OR for the conversion of kinetic energy into thermal energy.

## Key Points

- Grad P inside plasma outflow region reduces kinetic energy and enhances thermal energy
- Wake region and TCR must be considered for full energy balance
- Reconnection acts as a refrigerator

## 1 Introduction

Magnetic reconnection is seen as the responsible process for huge energy releases in the universe, such as during solar flares or in the context of geomagnetic storms and substorms. Reconnection leads to the conversion of previously stored magnetic energy into kinetic and thermal plasma energies and a topological reconfiguration of magnetic field lines. In the course of reconnection previously separated magnetic field lines get reconnected and removed from the initial reconnection site together with accelerated and heated plasma. Hence, magnetic reconnection is an important initiator process for the transport of mass, momentum, flux, and energy.

In order to obtain sufficiently large reconnection rates to explain the energy release rates in solar flares, *Petschek* [1964] introduced an analytical solution of the reconnection problem based on a small diffusion region—where the frozen‐in constraint is not valid—and the implementation of shocks. Slow‐mode shocks were observed in the distant tail beyond −70 *R*_*E*_ as boundaries of the tail lobe and the plasma sheet as well as on the front side of plasmoids [*Saito et al.*, 1995]. In the near‐Earth region (*X*≈−19 *R*_*E*_) the formation of slow shocks bounding the outflow region during an isolated substorm was confirmed by *Eriksson et al.* [2004] for a tailward propagating flow burst but failed for the earthward moving flow. This might be due to the asymmetric boundary conditions in the Earth's magnetosphere, i.e., the strong earthward directed magnetic field gradient. While shocks may freely evolve and propagate in the downtail direction, the presence of the strong dipole‐like inner magnetosphere forms a natural boundary for the establishment and propagation of shocks in the near‐Earth region. Alternatively, particle‐in‐cell simulations suggest that the outflow region of reconnection‐accelerated plasma is bounded by a pair of compound slow shocks/rotational discontinuity waves, rather than a pair of switch‐off slow shocks [*Liu et al.*, 2011a, 2011b]. In previous years, the Petschek model has been adapted and modified, allowing also considerations under a time‐dependent reconnection rate [*Semenov et al.*, 1983; *Biernat et al.*, 1987; *Semenov et al.*, 2004a; *Kiehas et al.*, 2009a].

Reconnection was shown to work quasi‐steady under certain conditions in certain environments, such as the solar wind and the magnetopause [e.g., *Mozer and Retino*, 2007; *Phan et al.*, 2006, 2013], where a steady state model can be used to describe the reconnection process. However, reconnection during magnetospheric substorms appears to work impulsively and rapidly [e.g., *Sergeev et al.*, 1987; *Angelopoulos et al.*, 1997]. Hence, we consider a time‐dependent model to be more applicable for substorm conditions. In the following we want to point out the advantages of a time‐dependent model over a steady state approach for magnetotail reconnection. Using a time‐dependent model allows us to compare the energy situation in a certain spatial region before and after reconnection, which is naturally not possible in steady state models, where reconnection never stops. Furthermore, the general topology implemented in the time‐dependent model incorporates important features that were observed in the magnetosphere but cannot be modeled in steady state reconnection. Amongst these features is the outflow region (OR), consisting of accelerated and heated plasma. While the OR remains permanently attached to the diffusion region in steady state reconnection, it detaches from the reconnection site in time‐dependent reconnection after reconnection ceased, transporting heated and accelerated plasma, energy, and reconnected magnetic flux. The OR corresponds to bursty bulk flows [*Angelopoulos et al.*, 1992], observed in the Earth's magnetotail. Since such a detached region of accelerated plasma is not implemented in steady state reconnection, its interplay with the surrounding medium can only be understood from time‐dependent models. Another feature, present in a time‐dependent model, but not in steady state reconnection, is the region of compressed field lines above and below the OR. Because the geometry in steady state reconnection remains an *X*‐type geometry with an OR confined by steady standing shocks, magnetic field lines above and below this steady OR do not get compressed. In a time‐dependent model, the ORs grow in size as more and more plasma gets added to this region and consequently compress the magnetic field lines above and below. These regions of compressed magnetic field lines above and below the ORs travel together with the ORs after reconnection stopped. In the Earth's magnetotail these regions are frequently observed and known as traveling compression regions (TCRs) [*Slavin et al.*, 1984]. Another domain, absent in steady state reconnection, but implemented in time‐dependent models, is the wake of the outflowing plasma. This region is absent in steady state reconnection because the OR remains attached to the diffusion region in these models, which does not allow a wake to develop behind the region of accelerated plasma. In time‐dependent reconnection, the OR detaches from the initial reconnection site after reconnection stopped, forming a wake in its trail. Furthermore, energy conversion continues at the fronts of the OR after reconnection ceased. This feature is naturally absent in steady state reconnection but observed in the magnetotail [*Angelopoulos et al.*, 2013]. All three regions (OR, TCR, and wake) and their interplay are important for energy considerations, as outlined in this paper. Due to the absence of wakes, TCRs, detached ORs, and postreconnection energy conversion in steady state models, a treatment of the reconnection problem via a time‐dependent model is crucial.

The following investigations are based on a time‐dependent Petschek‐type magnetic reconnection model, first developed by *Semenov et al.* [1983] and *Biernat et al.* [1987]. A more recent description is given in *Kiehas et al.* [2009a]. Further applications of the model for the compressible case show strong plasma compression ahead of the OR [*Semenov et al.*, 1998a], a variety of MHD waves and shocks [*Heyn et al.*, 1988], and its validity in the fast reconnection regime [*Erkaev et al.*, 2001]. The model has been extended for asymmetric reconnection [*Heyn et al.*, 1988; *Semenov et al.*, 1992] and has been applied to magnetopause [e.g., *Biernat et al.*, 1998] and magnetotail reconnection [e.g., *Semenov et al.*, 2005; *Kiehas et al.*, 2009a]. The model has been used to obtain information about the reconnection process, such as the reconnection rate, reconnected magnetic flux, or the location of the reconnection site [*Semenov et al.*, 2005; *Ivanova et al.*, 2007; *Kiehas et al.*, 2008]. The modeled large‐scale disturbances have been shown to agree well with observations [*Kiehas et al.*, 2009a] and match those obtained from simulations [*Ugai and Zheng*, 2006a, 2006b].

**Figure 1:**
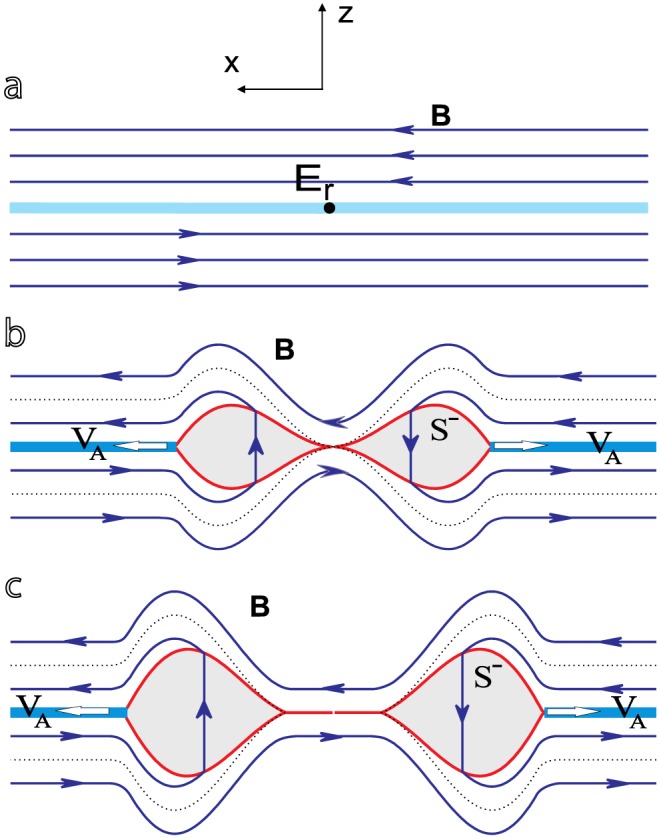
Time‐dependent Petschek reconnection. (a–c) The evolution of the plasma outflow regions/shock structures and the change in magnetic field topology. Reconnection is initiated at the origin of the sketched coordinate system. The light blue line denotes the current sheet, separating two antiparallel magnetic fields (blue arrows). Inside a locally confined region, a pulsative, time‐varying reconnection electric field *E*_*r*_ is established (Figure 1a), leading to the acceleration of plasma in opposite directions along the current sheet. Due to the temporally restricted activity of *E*_*r*_, the plasma outflow is confined to closed outflow regions (grey areas, Figure 1b). These regions are bounded by shocks (red) and detach from the initial reconnection site after *E*_*r*_ vanishes (Figure 1c). Since magnetic field lines from both sides of the current sheet are connected via the outflow regions, reconnected magnetic flux is transported together with the plasma outflow regions. The dotted lines represent the separatrices [after *Semenov et al.*, 2004b].

**Figure 2:**
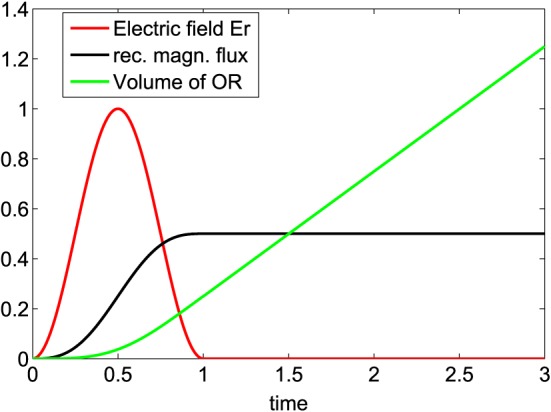
Normalized reconnection electric field (red, modeled as $E=\varepsilon \;E_{A}\;\sin^{2}(\mathrm{\pi t}/T_{0})$ and active during 0 < *t*≤*T*_0_with *T*_0_=1), normalized reconnected flux (black), and volume of the outflow region (per unit length of the reconnection line, green). The reconnection electric field, reconnected magnetic flux, and volume of the OR are normalized to the maximum value of the reconnection electric field (*ε* *E*_*A*_), *c* *ε* *E*_*A*_ *T*_0_, and $\varepsilon \;v_{A}\;B_{0}\;T_{0}^{2}$, respectively. Time is normalized to *T*_0_.

The basic concept of the time‐dependent reconnection model used in this work is shown in Figure 1. Initially, a current sheet, modeled as tangential discontinuity, separates two antiparallel magnetic fields, which are embedded in two identical, uniform, and compressible plasmas. At some point reconnection is initiated in a localized region of the current sheet, the diffusion region, by processes that cannot be described by ideal MHD. However, the large‐scale energy conversion and redistribution takes primarily place in the convective region surrounding the diffusion region. Consequently, we will not discuss the nature of the dissipation process inside the diffusion region, resulting in the generation of the reconnection electric field. Instead, we include all possible dissipation scenarios by defining an a priori reconnection electric field (*E*_*r*_(*t*) in Figure 1a) as arbitrary function of time and consider *E*_*r*_(*t*) as initial condition. The unsteady, impulsive behavior of the reconnection process is reflected in the time‐varying appearance of *E*_*r*_(*t*). The reconnection electric field rises in time to a certain maximum and falls down thereafter (see Figure 2).

While magnetic field lines from opposite sides of the current sheet are connected via standing shocks in the steady state Petschek model, the time‐varying reconnection electric field in our model leads to the formation of enclosed plasma outflow regions (ORs) over which magnetic field lines are connected (Figure 1b). After reconnection stopped, the outflow regions are no longer connected to the initial reconnection site and they propagate in opposite directions along the current sheet (Figure 1c). At this stage no more reconnected flux is added to the system, but the volume of the OR grows during their propagation as more and more plasma gets accelerated over the shocks and energy conversion continues (see Figure 2).

Energy partition in magnetotail reconnection was studied by *Eastwood et al.* [2013], based on 18 ion diffusion region encounters by Cluster. Calculations of the energy fluxes showed that the ion enthalpy flux is the dominant component of the energy fluxes. In a global MHD simulation of magnetotail reconnection, *Birn and Hesse* [2005] discussed the changes, transport, and conversion of magnetic, thermal, and kinetic energies at an advanced stage of reconnection, when the plasmoid has moved downtail already. Their simulation showed that Poynting flux is converted at slow shocks into kinetic energy flux, most of which is immediately transferred to enthalpy flux. Hence, kinetic energy acts as a mediator in the conversion of magnetic into thermal energy. Without distinguishing the aforementioned regions (OR, TCR, and wake), their results clearly show the decrease in magnetic energy in the inflow region and increase in thermal and kinetic energies in the outflow region. *Yamada et al.* [2014] studied the conversion of magnetic energy during reconnection in a laboratory plasma and found that 50% of the magnetic energy gets converted into particle thermal and kinetic energies. In agreement with the *Birn and Hesse* [2005] and *Eastwood et al.* [2013] studies, they also found a dominance of the thermal energy over the kinetic flow energy. Together with *Phan et al.* [2013], *Eastwood et al.* [2013], and *Shay et al.* [2014], their study also showed that only a small percentage of the inflowing magnetic energy gets converted into electron thermal energy. In this work, we want to address the transfer of magnetic, thermal, and kinetic energies amongst the three regions formed by reconnection (OR, wake, and TCR). Using a time‐dependent reconnection model allows us to include the TCR and assess the energy changes and transfers also in that region.

## 2 Overview of the Analytical Model

The time‐dependent reconnection model is described in detail in *Biernat et al.* [1987], *Semenov et al.* [2004b], and *Kiehas et al.* [2009a]. In this paper, we only give a short overview of the model and restrict the description to those parts that are relevant for this publication. For convenience of calculations, all equations are written in CGS units throughout this work.

The two initial antiparallel magnetic fields on either side of the tangential discontinuity are represented in the form **B**_1_ = −**B**_2_ = (*B*_0_, 0), with *B*_0_ as background magnetic field. If the reconnection electric field *E*_*r*_ is much smaller than the Alfvén electric field, $E_{A}=\frac{1}{c}v_{A}B_{0}$, where *v*_*A*_ and *B*_0_ denote the Alfvén velocity and the background magnetic field, respectively, a small parameter *ε* can be introduced

$$\varepsilon \equiv \frac{c\;E_{r}}{v_{A}\;B_{0}}\ll 1.$$

In this case, the outflow regions can be assumed as thin boundary layers, which allows a perturbation analysis of the MHD equations, with *ε* as small expansion parameter. In general, information about all variables in the MHD equation set can be summarized in one state vector **U** = **U**(*ρ*, *p*, **B**, **v**) [*Rijnbeek and Semenov*, 1993]. We restrict ourselves to the expansion of the magnetic field only:

(1) $$B=B^{\left(0\right)}+\varepsilon B^{\left(1\right)}+\varepsilon^{2}B^{\left(2\right)}+...$$

where **B** ^(0)^ denotes undisturbed quantities and **B** ^(1)^ (**B** ^(2)^) disturbances of the first (second) order. While quantities tangential to the current sheet are of the order of ∼1, perpendicular components are of the order of ∼*ε*, as can be shown by an order of magnitude estimation. With this, the outflow regions can be assumed to behave as thin boundary layers where the tangential and normal components correspond approximately to *x* and *z* components, respectively. In their general form, the magnetic fields in the inflow and outflow regions can be written as

(2) $$B=\left(B_{0}+B_{x}^{\left(1\right)},B_{z}^{\left(1\right)}\right),$$

(3) $$\tilde{B}=\left(0,{\tilde{B}}_{z}^{\left(1\right)}\right),$$

where **B** and $\tilde{B}$ represent the magnetic fields in the inflow and outflow regions, respectively.

For the first quadrant one finds for the magnetic field, pressure, and velocity components inside the OR [*Semenov et al.*, 2004b; *Kiehas et al.*, 2009a],

(4) $${\tilde{B}}_{x}=0,$$

(5) $${\tilde{B}}_{z}=\frac{c}{v_{A}}E_{r}(t-x/v_{A}),$$

(6) $$\tilde{p}=p_{0}+\frac{B_{0}^{2}}{8\;\pi },$$

(7) $${\tilde{v}}_{x}=v_{A},$$

(8) $${\tilde{v}}_{z}=0,$$

where $E_{r}\left(t-x/v_{A}\right)$ denotes the reconnection electric field. It must be noted that we use unnormalized quantities throughout this work, contrary to previous publications [*Semenov et al.*, 2004b; *Kiehas et al.*, 2009a, 2009b]. For the solutions, presented in equations (4), (5), (6), (7), (8), we disrupt expansion (1) after first‐order terms and neglect higher‐order terms. With this, and assuming a homogeneous background plasma density distribution, we find for the outflow velocity ${\tilde{v}}_{x}$ the Alfvén velocity *v*_*A*_. The shape of the OR, defining the location of the OR in the *x*‐*z* plane, can be found as [*Biernat et al.*, 1987]

(9) $$z=f(x)=\frac{\rho_{0}}{\tilde{\rho }}\frac{c}{v_{A}B_{0}}\;x\;E_{r}(t-x/v_{A}),$$

where *ρ*_0_ and $\tilde{\rho }$ denote the plasma density in the inflow and outflow regions, respectively, with

(10) $$\tilde{\rho }=\rho_{0}\frac{\gamma (\beta +1)}{\gamma (\beta +1)-1},$$

where $\gamma =\frac{c_{P}}{c_{V}}$ and $\beta =\frac{8\;\pi \;p_{0}}{B_{0}^{2}}$ denote the polytropic index and plasma *β*, respectively. For an ideal monoatomic (*γ* = 5/3), magnetic field dominated ( β→0) plasma, the compressibility coefficient yields $\frac{\rho_{0}}{\tilde{\rho }}=2/5$.

It should be noted that $\tilde{\rho }$, $\tilde{p}$, and ${\tilde{v}}_{x}$ do not depend on the reconnection rate. On the other hand, ${\tilde{B}}_{z}$ and the shape of the OR (equations (5) and (9)) do depend on the reconnection rate. Consequently, a stronger reconnection rate does not lead to a stronger acceleration, but to an increase in the size of the OR.

### 2.1 The Volume of the OR

Throughout this paper, all calculations are performed for the first quadrant only (cf. Figure 1, with the center of the coordinate system at the reconnection site), due to the symmetry of the problem.

Under the idealized model assumptions, the leading front of the OR propagates with Alfvén speed. Consequently, the leading front can be located at *x* = *v*_*A*_ *t*. With this, the volume of the OR can be derived by integrating the function *f* from equation (9) over *x* with the lower (upper) boundary *x* = 0 (*x* = *v*_*A*_ *t*). For the first quadrant (*Q*1), this yields

(11) $$V_{\mathrm{OR}}^{Q1}=\frac{\rho_{0}}{\tilde{\rho }}\frac{c}{v_{A}B_{0}}\overset{v_{A}t}{\underset{0}{\int }}x\;E_{r}\left(t-x/v_{A}\right)\;dx.$$

We can define the functions $F(t)=c\;\overset{t}{\underset{0}{\int }}E_{r}(\tau )\;d\tau$ and $G(t)=\overset{t}{\underset{0}{\int }}F(\tau )\;d\tau$ which are proportional to the reconnected magnetic flux and the volume of the OR, shown in Figure 2. In the following we show the derivation of the volume of OR in dimensional units. During the active phase of reconnection, the reconnected magnetic flux *F*(*t*) builds up and reaches its maximum level *F*_0_ after reconnection ceased. Then, the volume of the OR increases linearly with time. From equation (11) one gets after changing the integration variable *τ* = *t* − *x*/*v*_*A*_,

$$V_{\mathrm{OR}}^{Q1}=\frac{\rho_{0}}{\tilde{\rho }}\frac{c}{v_{A}B_{0}}\overset{t}{\underset{0}{\int }}{v_{A}}^{2}(t-\tau )E_{r}(\tau )d\tau ,$$

$$V_{\mathrm{OR}}^{Q1}=\frac{\rho_{0}}{\tilde{\rho }}\frac{v_{A}}{B_{0}}\left[(t-\tau )F(\tau ){|}_{0}^{t}+\overset{t}{\underset{0}{\int }}F(\tau )d\tau \right],$$

where the term (*t* − *τ*) is zero for the upper boundary *τ* = *t* and the term *F*(*τ*) is zero for the lower boundary *τ* = 0. With this, the volume of the OR in the first quadrant can be written as

(12) $$V_{\mathrm{OR}}^{Q1}=\frac{\rho_{0}}{\tilde{\rho }}\frac{v_{A}}{B_{0}}\;G(t).$$

As can be seen from Figure 2, after *t* = *T*_0_ = 1, the reconnection electric field drops to zero, the reconnected magnetic flux reaches its constant level *F*_0_, and *G*(*t*) increases linearly. Consequently, for *t* ≫ *T*_0_ the function *G*(*t*) can be approximated as *G*(*t*) = *t* *F*_0_. With this, the volume of the OR increases linearly with time.

## 3 Change of Kinetic Energy Inside the OR

The kinetic energy of the plasma inside the OR is given as

$$W_{k}=\frac{1}{2}\;\tilde{\rho }\;v_{A}^{2}\;\underset{\mathrm{OR}}{\int }\;dV.$$

With $v_{A}=\frac{B_{0}}{\sqrt{4\;\pi \;\rho_{0}}}$ and the integral over the OR from equation (12), this yields

(13) $$W_{k}^{\mathrm{OR},Q1}=\frac{v_{A}\;B_{0}}{8\pi }\;G(t).$$

For *t* ≫ *T*_0_, one gets

(14) $$W_{k}^{\mathrm{OR},Q1}=\frac{v_{A}\;B_{0}}{8\pi }\;t\;F_{0}.$$

Since equation (13) is independent from the compressional factor $\frac{\rho_{0}}{\tilde{\rho }}$, the kinetic energy inside the outflow region is the same for the incompressible and compressible cases. This can be understood by the balance of two contributions to the kinetic energy: In the compressible case, the density inside the outflow region is enhanced compared to the incompressible case, leading to a higher kinetic energy content. On the other hand, the volume of the outflow region is reduced in the compressible case by the factor $\frac{\rho_{0}}{\tilde{\rho }}$, leading to smaller kinetic energy content. These two effects balance each other, yielding together no change in the kinetic energy content of the outflow region when generalizing the problem from the simplified incompressible situation to the compressible case.

## 4 Change of Magnetic Energy Inside the OR

Considering an area in space with the volume of the OR in the first quadrant, the change in the magnetic energy inside this region before and after reconnection is

(15) $$\Delta W_{B}^{\mathrm{OR},Q1}=\underset{\mathrm{OR},Q1}{\int }\left(\frac{{\left({\tilde{B}}_{z}^{\left(1\right)}\right)}^{2}}{8\pi }-\frac{B_{0}^{2}}{8\pi }\right)\;dV.$$

Since ${\left({\tilde{B}}_{z}^{\left(1\right)}\right)}^{2}$ corresponds to a second‐order term, the magnetic energy inside the OR after reconnection can be neglected compared to the magnetic energy inside an area corresponding to the same volume as the OR before reconnection. Hence, the change of the magnetic energy inside the OR in the first quadrant due to reconnection can be written as

(16) $$\Delta W_{B}^{\mathrm{OR},Q1}=-\frac{B_{0}^{2}}{8\pi }\underset{\mathrm{OR},Q1}{\int }\;dV.$$

With the volume of the OR from equation (12), we find

(17) $$\Delta W_{B}^{\mathrm{OR},Q1}=-\frac{v_{A}\;B_{0}}{8\pi }\frac{\rho_{0}}{\tilde{\rho }}\;G(t).$$

Due to the compressional factor $\frac{\rho_{0}}{\tilde{\rho }}$ in equation (17), the change in the magnetic energy in the OR is reduced for compressible situations compared to the incompressible case. For β→0 and *γ* = 5/3, the magnetic energy in the OR is reduced for the factor 2/5 compared to the incompressible case. This effect is due to the reduced volume of the OR in the compressible case, which is not compensated by other effects, as it is the case for the kinetic energy.

While the amount of kinetic energy (equation (13)) and decrease in magnetic energy inside the OR (equation (17)) are balanced in the incompressible case ( $\rho_{0}=\tilde{\rho }$), we find a relation between the kinetic and magnetic energies inside the OR in the first quadrant for the compressible case:

(18) $$W_{k}^{\mathrm{OR},Q1}=-\frac{\tilde{\rho }}{\rho_{0}}\Delta W_{B}^{\mathrm{OR},Q1}.$$

Hence, the decrease in the magnetic energy does not fully compensate the increase in the kinetic energy inside the OR. In other words, not all of the kinetic energy is due to the direct conversion of magnetic energy into kinetic energy; the decrease in the magnetic energy inside the OR is not sufficient to obtain the necessary amount of kinetic energy.

## 5 Change of Thermal Energy Inside the OR

Analogous to the considerations for the change of the magnetic energy inside the OR, the change of the thermal energy inside the OR in the first quadrant before and after reconnection is

(19) $$\Delta W_{T}^{\mathrm{OR},Q1}=\frac{1}{\gamma -1}\underset{\mathrm{OR},Q1}{\int }\left(\tilde{p}-p_{0}\right)\;dV.$$

Replacing $\tilde{p}$ from the relation of pressure balance between the IR and OR regions, equation (6), it follows

(20) $$\Delta W_{T}^{\mathrm{OR},Q1}=\frac{1}{\gamma -1}\frac{{B_{0}}^{2}}{8\;\pi }\underset{\mathrm{OR},Q1}{\int }\;dV.$$

With equation (12), this yields

(21) $$\Delta W_{T}^{\mathrm{OR},Q1}=\frac{v_{A}\;B_{0}}{8\;\pi }\frac{\rho_{0}}{\tilde{\rho }}\frac{1}{\gamma -1}G(t).$$

From equations (13), (17), and (21), we see

(22) $$\Delta W_{B}^{\mathrm{OR}}<\Delta W_{T}^{\mathrm{OR}}+W_{k}^{\mathrm{OR}}.$$

Hence, the amount of kinetic and thermal energies inside the OR is not balanced by the decrease of magnetic energy inside the OR. Since the decrease of magnetic energy is not sufficient to feed the increase in the kinetic and thermal energies, other regions must contribute to the acceleration and heating of plasma. To identify these regions, we extend the analysis beyond the OR.

## 6 The Magnetic Energy in the Inflow Region

Before reconnection starts, the magnetic energy density in the surrounding medium is given by ${B_{0}}^{2}/8\pi$. Reconnection disturbs the medium and magnetic field, which leads to a magnetic field **B**_after_. The change in the magnetic energy, resulting from this disturbance in the medium, can be written as difference of the magnetic energy after (related to *B*_after_) and before reconnection (related to *B*_0_),

(23) $$\Delta W_{B}=\int \left(\frac{\overset{2}{\underset{\mathrm{after}}{B}}}{8\pi }-\frac{{B_{0}}^{2}}{8\pi }\right)\;dV.$$

Inserting for **B**_after_ from equation (2) and neglecting terms of the second order, the magnetic energy appears in our 2‐D configuration as

(24) $$\Delta W_{B}=\frac{B_{0}}{4\pi }\int \int \overset{\left(1\right)}{\underset{x}{B}}dxdz.$$

By introducing a vector potential **A** = (0,*A*,0) of the form **B** = ∇×**A**, the components of the magnetic field can be written in terms of *A*,

(25) $$B_{x}^{\left(1\right)}=-\frac{\mathrm{\partial A}}{\mathrm{\partial z}},\;B_{z}^{\left(1\right)}=\frac{\mathrm{\partial A}}{\mathrm{\partial x}}.$$

With this, the change in the magnetic energy can be displayed by the integral over a vector potential,

$$\Delta W_{B}=-\frac{B_{0}}{4\pi }\int \int \frac{\mathrm{\partial A}}{\mathrm{\partial z}}dx\;dz,$$

$$\Delta W_{B}=-\frac{B_{0}}{4\pi }\int \left(A{|}_{z=\infty }-A{|}_{z=z_{0}}\right)dx.$$

Considering a thin boundary layer analysis, we can assume that *z*_0_ = 0, since any error occurring out of this assumption is of the second order. Since the magnetic potential **A** is defined to be zero at infinity, this leads to [see also *Semenov et al.*, 1998b]

(26) $$\Delta W_{B}=\frac{B_{0}}{4\pi }\int A{|}_{z=0}dx,$$

where *A*|_0_ can be expressed via $B_{z}^{\left(1\right)}{|}_{0}$ from equation (25),

(27) $$A{|}_{0}=\int \overset{\left(1\right)}{\underset{z}{B}}{|}_{z=0}dx.$$

**Figure 3:**
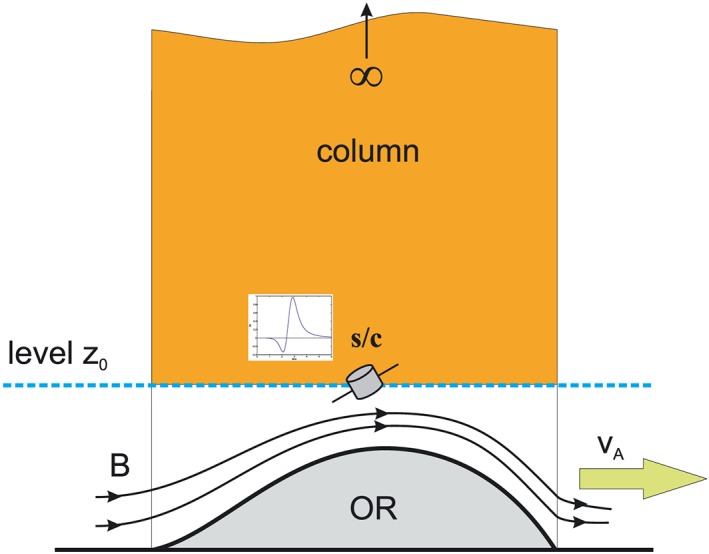
Column containing enhanced amount of magnetic energy, which can be obtained by equations (26) and (27). By measuring the magnetic field disturbances at the level *z* = 0, the amount of magnetic energy in the column above can be obtained [after *Kiehas et al.*, 2009b].

Hence, the disturbances $B_{z}^{\left(1\right)}$, measured at the level *z* = 0, give the change in the magnetic energy in a column *z* > 0 by double integration of $B_{z}^{\left(1\right)}$ and the change in the magnetic energy in the entire column can be calculated (see Figure 3).

Analogous to the incompressible case [*Kiehas et al.*, 2009b], we can find the vector potential at *z*_0_ as

(28) $$A{|}_{0}=\frac{c}{v_{A}}\left(-\frac{v_{A}}{c}\;F\left(t-\frac{x}{v_{A}}\right)+\frac{\rho_{0}}{\tilde{\rho }}\;x\;E_{r}\left(t-\frac{x}{v_{A}}\right)\right).$$

Inserting the potential at *z* = *z*_0_ = 0 from equation (28) in equation (26), we find the change in the magnetic energy inside the inflow region (*x*:[0,*x*]; z:[0, ∞)) as function of *x*:

(29) $$\Delta W_{B}^{\mathrm{IR},Q1}(x)=\frac{B_{0}}{4\pi }\left[v_{A}\;\left(1-\frac{\rho_{0}}{\tilde{\rho }}\right)G\left(t-\frac{x}{v_{A}}\right)-\frac{\rho_{0}}{\tilde{\rho }}\;x\;F\left(t-\frac{x}{v_{A}}\right)-v_{A}\left(1-\frac{\rho_{0}}{\tilde{\rho }}\right)\;G(t)\right].$$

For the inflow region between the initial reconnection site and the leading edge of the OR (0 < *x* < *v*_*A*_ *t*), we find

(30) $$\Delta W_{B}^{\mathrm{IR},Q1}(x=v_{A}\;t)=-\frac{v_{A}\;B_{0}}{4\pi }\left(\frac{\rho_{0}}{\tilde{\rho }}\right)\;G(t).$$

**Figure 4:**
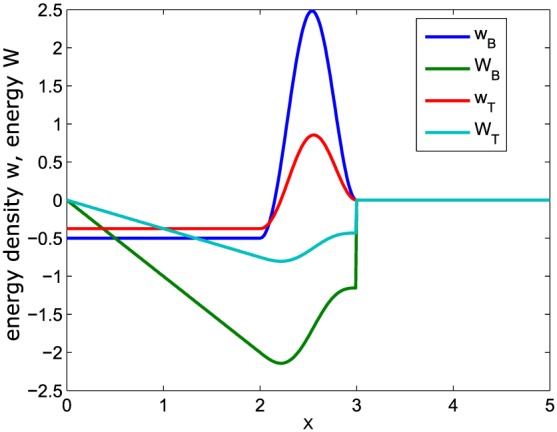
Change of normalized magnetic energy *W*_*B*_ (green) and thermal energy *W*_*T*_ (light blue) as function of *x* for *t* = 3 in the inflow region. The initial reconnection site is at *x* = 0 and the location of the OR between 2 < *x* < 3. *w*_*B*_ and *w*_*T*_ denote the corresponding normalized energy densities. *x*, *w*, and *W* are normalized to *v*_*A*_ *T*_0_, *c* *ε* *E*_*A*_ *T*_0_, and $\frac{B_{0}^{2}}{8\pi }\;\varepsilon {\left(v_{A}\;T_{0}\right)}^{2}$, respectively.

This function decreases linearly from zero at the reconnection site (*x* = 0) to a negative value near the trailing edge of the OR region and then increases in the region above the OR (green curve in Figure 4).

For a better understanding of the energy redistribution, let us split the inflow region into two different parts: the wake region behind the OR (0 < *x* < *v*_*A*_(*t* − *T*_0_)) and the region of compressed magnetic field lines above the OR, corresponding to a TCR (*v*_*A*_(*t* − *T*_0_) < *x* < *v*_*A*_ *t*). For each of these regions we find for *t* ≫ *T*_0_ inside the wake region,

(31) $$\Delta W_{B}^{\mathrm{wake},Q1}=-\frac{v_{A}\;B_{0}}{4\pi }\;t\;F_{0},$$

and inside the TCR

(32) $$\Delta W_{B}^{\mathrm{TCR},Q1}=\frac{v_{A}\;B_{0}}{4\pi }\left(\frac{\rho_{0}}{\tilde{\rho }}\right)\;t\;F_{0}.$$

Equation (31) shows a decrease of magnetic energy inside the wake region, while equation (32) shows an increase inside the TCR, as can also be seen from Figure 4. The decrease of Δ*W*_*B*_ in the wake can be understood in terms of the reconfiguration of magnetic field lines in the course of reconnection. The wake corresponds to the inflow region where field lines get disconnected and are finally reconnected via the OR to field lines from the other hemisphere and transported away from the reconnection site. This depletion of magnetic flux and consequently magnetic energy is expressed by the decrease of Δ*W*_*B*_ in the wake. Inside the TCR field lines are compressed due to the appearance of the OR. This enhanced field line density is reflected in the increase of Δ*W*_*B*_ in the TCR. For the incompressible case (*ρ*_0_= $\tilde{\rho }$), we find $\Delta W_{B}^{\mathrm{TCR}}=-\Delta W_{B}^{\mathrm{wake}}$. Hence, the decrease of Δ*W*_*B*_ in the wake is fully compensated by an increase in the TCR. In the compressible situation, $\Delta W_{B}^{\mathrm{TCR}}$ is smaller than $\Delta W_{B}^{\mathrm{wake}}$ for the factor $\frac{\rho_{0}}{\tilde{\rho }}$. Physically, this can be explained by the following: In a compressible plasma situation, the OR gets compressed. Due to this reduction of its height (relative to incompressible situations), disturbances in the TCR are reduced too. Consequently, the magnetic energy transported inside the TCR is reduced in compressible plasma situations.

With this, not all of the additional magnetic energy inside the TCR is redistributed from magnetic energy loss inside the wake. Thermal energy needs to be considered in this case, which is demonstrated in section 7.

Let us compare the increase in Δ*W*_*B*_ in the TCR with the kinetic energy inside the OR. For the incompressible case, equations (14) and (32) show that the increase in Δ*W*_*B*_ inside the TCR is twice as much as the kinetic energy inside the OR. For the compressible case, this relation is decreased for the compressible factor $\frac{\rho_{0}}{\tilde{\rho }}$,

(33) $$\Delta W_{B}^{\mathrm{TCR},Q1}=2\frac{\rho_{0}}{\tilde{\rho }}W_{k}^{\mathrm{OR},Q1}.$$

Consequently, the magnetic energy transported inside a TCR corresponds to 80 to 200% of the kinetic energy inside the OR, depending on the plasma compressibility.

## 7 The Thermal Energy in the Inflow Region

Analogous to the change in the magnetic energy inside the IR, we can derive the change in the thermal energy inside the IR from the difference in the energies before and after reconnection:

(34) $$\Delta W_{T,Q1}=\frac{1}{\gamma \;-1}\underset{\mathrm{IR},Q1}{\int }\left(\left(p_{0}+p^{\left(1\right)}-p_{0}\right)\right)dxdz\;=\frac{1}{\gamma \;-1}\underset{\mathrm{IR},Q1}{\int }p^{\left(1\right)}\;dxdz.$$

With $p^{\left(1\right)}=\gamma \frac{p_{0}}{\rho_{0}}\rho^{\left(1\right)}$ and $\rho^{\left(1\right)}=-\rho_{0}\;\mathrm{div}\;\xi$, where ***ξ*** denotes the displacement vector (see Appendix B), this yields with Gauss' theorem

(35) $$\Delta W_{T,Q1}=-\frac{\gamma \;p_{0}}{\gamma \;-1}\int \mathrm{div}\xi dxdz\;=\frac{\gamma \;p_{0}}{\gamma \;-1}\overset{\infty }{\underset{0}{\int }}\xi_{z}(t,x,0)dx.$$

With equation (B5) and $p_{0}=\frac{\beta \;B_{0}}{8\;\pi }$ we find the change in the thermal energy inside the inflow region (*x*:[0,*x*]; *z*:[0, ∞)) as function of *x*:

(36) $$\Delta W_{T}^{Q1}(x)=\frac{\gamma \;\beta }{\gamma \;-1}\frac{B_{0}}{8\;\pi }\left[v_{A}\;\left(1-\frac{\rho_{0}}{\tilde{\rho }}\right)G\left(t-\frac{x}{v_{A}}\right)-\frac{\rho_{0}}{\tilde{\rho }}\;x\;F\left(t-\frac{x}{v_{A}}\right)-v_{A}\left(1-\frac{\rho_{0}}{\tilde{\rho }}\right)\;G(t)\right].$$

For the inflow region between the initial reconnection site and the leading edge of the OR (0 < *x* < *v*_*A*_ *t*), we find

(37) $$\Delta W_{T}^{\mathrm{IR},Q1}(x=v_{A}\;t)=-\frac{\gamma \;\beta }{\gamma \;-1}\frac{v_{A}\;B_{0}}{8\;\pi }\left(1-\frac{\rho_{0}}{\tilde{\rho }}\right)\;G(t).$$

By comparing equations (37) and (30), we see that the functions describing the distribution of magnetic and thermal energy in the inflow region behave identical and differ only by a constant factor.

As change in the thermal energy inside the wake region we find,

(38) $$\Delta W_{T}^{\mathrm{wake},Q1}=-\frac{\gamma \;\beta }{\gamma \;-1}\frac{v_{A}\;B_{0}}{8\pi }\;t\;F_{0},$$

and inside the TCR

(39) $$\Delta W_{T}^{\mathrm{TCR},Q1}=\frac{\gamma \;\beta }{\gamma \;-1}\frac{\rho_{0}}{\tilde{\rho }}\frac{v_{A}\;B_{0}}{8\pi }\;t\;F_{0}.$$

Hence, the thermal energy inside the inflow region decreases. For the two areas of the IR, the thermal energy behaves differently. While it decreases in the wake, it increases inside the TCR. Physically, this can be understood by the following: The TCR corresponds to a compression of magnetic field lines and plasma, which leads to an increase in the magnetic and thermal energies therein. The wake region, on the other hand, corresponds to a rarefaction region. Consequently, the magnetic and thermal energies decrease. With the decrease of the thermal energy in the wake of the OR, reconnection effectively acts as a refrigerator, where cooled plasma is left behind in the wake of the OR.

## 8 Total Energy Budget

**Figure 5:**
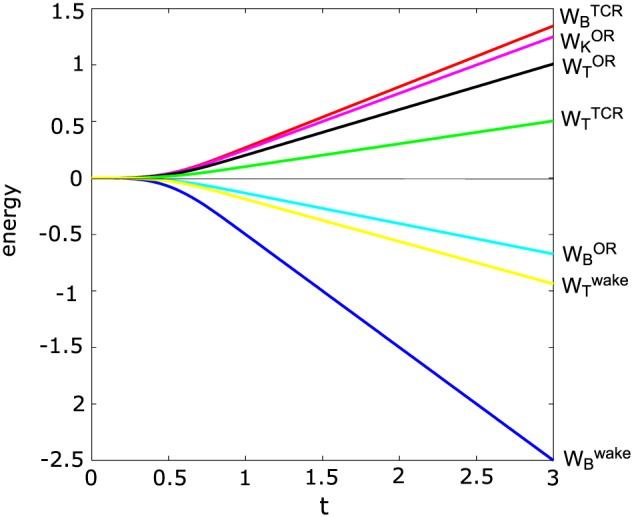
Temporal evolution of each energy form (kinetic, thermal, and magnetic) in the three different regions (OR, wake, and TCR) in the theoretical model. Calculations are done for *β* = 0.15. Time and energies are normalized to *T*_0_ and $\frac{B_{0}^{2}}{8\pi }\;\varepsilon {\left(v_{A}\;T_{0}\right)}^{2}$, respectively.

The relation between each energy component (kinetic, thermal, and magnetic) in the three different regions (OR, wake, and TCR) is shown in Figure 5.

The overall energy conversion can be described as follows. During the reconnection process plasma streams toward the reconnection site in the inflow region, gets heated and accelerated and leaves the scene via the outflow regions. Due to the reconfiguration of magnetic field lines, the magnetic energy inside the OR is considerably reduced (equation (17)). However, this reduction is not sufficient to explain the total increase in the kinetic and thermal energies in the OR (equation (22)). Consequently, energy from the inflow region is redistributed into the outflow region to support the heating and acceleration of plasma.

The total energy balance can be found as

(40) $$W_{k}^{\mathrm{OR},Q1}+W_{T}^{\mathrm{OR},Q1}+\Delta W_{B}^{\mathrm{OR},Q1}+\Delta W_{B}^{\mathrm{IR},Q1}+\Delta W_{T}^{\mathrm{IR},Q1}=0.$$

This is valid for an arbitrary electric field *E*_*r*_(*t*). Inserting each of these terms as derived in the previous sections, we see indeed that the total energy balance is zero. Hence, the energy is getting transferred solely in between these terms, assuming the dissipation terms inside the diffusion region to be small. As mentioned earlier, the IR can be divided into a wake and a TCR region for an impulsive reconnection electric field. While the energy in the wake region decreases, it increases inside the TCR. For the incompressible case, the changes in those regions balance each other. For compressible situations, however, one needs to take into account thermal energy and the decrease of energy in the wake, associated with an increase of energy in the TCR, to achieve full energy balance. The energy increase in the TCR can be understood with the OR as a transition region, whose expansion leads to an increase in thermal and magnetic energies inside the TCR.

It needs to be noted that the disturbances inside the OR are of the order of 1, while its size is of the order of *ε*. For the IR the situation is vice versa. Consequently, it is the relatively large area of the IR over which all the small disturbances get integrated and consequently cannot be neglected.

It can also be shown that the total energy carried by the plasma inside the OR is greater than the total amount of energy inside a TCR,

(41) $$W_{k}^{\mathrm{OR},Q1}+W_{T}^{\mathrm{OR},Q1}>\Delta W_{B}^{\mathrm{TCR},Q1}+\Delta W_{T}^{\mathrm{TCR},Q1}.$$

## 9 Two‐Dimensional MHD Simulations

Since the results so far are based on an idealized analytical model, we want to investigate how these idealizations, like an infinitely thin current sheet, affect the results. For this purpose, we run a 2‐D MHD simulation, which includes a finite current sheet thickness and compare the results on the energy budget with those from the analytical model. In the used simulation magnetic field and plasma parameters are assumed to satisfy the equations of resistive magnetohydrodynamics:

(42) $$\frac{\partial \rho v}{\mathrm{\partial t}}+\nabla \cdot \left(\rho vv+\Pi \;I-\frac{1}{4\pi }BB\right)=0,$$

(43) $$\Pi =p+B^{2}/(8\pi ),\;\frac{\partial \rho }{\mathrm{\partial t}}+\nabla \cdot (\rho v)=0,$$

(44) $$\frac{\partial }{\mathrm{\partial t}}\left(\rho v^{2}/2+\frac{1}{8\pi }B^{2}+\frac{1}{\gamma -1}p\right)+\nabla \cdot \left[v\left(\rho v^{2}/2+\frac{1}{4\pi }B^{2}+\frac{\gamma }{\gamma -1}p\right)-\frac{B(v\cdot B)}{4\pi }\right]=0,$$

(45) $$\frac{\partial B}{\mathrm{\partial t}}-\nabla \cdot \;(v\times B)+\nabla \times \;(\nu \;\nabla \times \;B)=0,$$

(46) ∇·B=0.

Here *ν* = *c* ^2^ *η*/(4*π*) is the magnetic viscosity, where *η* is the plasma resistivity; *I* and *Π* denote the unit matrix and total pressure (sum of the magnetic and plasma pressures), respectively, and *γ* is set equal to 5/3.

The electric field can be obtained from Ohm's law

(47) $$E=-\frac{1}{c}v\times B+\frac{c\;\eta }{4\pi }\nabla \times \;B.$$

Generally, the plasma resistivity can be a function of coordinates and time.

At the initial moment we assume the equilibrium current sheet with hyperbolic variation of the tangential magnetic field component

(48) $$B_{x}=B_{0}\tanh(z/\Delta ),\;\rho =\rho_{0},\;B_{z}=0.$$

Initially, the plasma is assumed to be in a rest with zero velocity components. Then we introduce a temporal and spatial variation of the resistivity,

(49) $$\eta =\eta_{0}+\eta_{1}t\exp(-\mathrm{at})\exp\left[-b\left({\left(x-x_{0}\right)}^{2}+z^{2}\right)\right],$$

where *η*_0_=0.001 is the background resistivity and the parameter *η*_1_=0.1 determines the amplitude of the resistivity pulse.

For numerical calculations we use a Godunov‐type [*Godunov*, 1959] finite volume scheme based on the approximate Riemann solver, which was proposed by *Powell* [1994] for ideal MHD equations. In our case we take into account a finite resistivity, and thus, we have a more complicated induction equation (45) for the magnetic field, which is of an elliptical type. We have implemented an iterative procedure to solve this equation at each time step.

The divergence‐free condition (46) is controlled by the method of projection, as outlined in *Toth* [2000].

Furthermore, for computational convenience we introduce dimensionless variables through

(50) $$\begin{array}{cc} \tilde{x} & =x/L_{0},\;\tilde{z}=z/L_{0},\;\tilde{t}=t/T_{0},\;\tilde{p}=4\mathrm{\pi p}/B_{0}^{2},\\ \tilde{\rho } & =\rho /\rho_{0},\;\tilde{B}=B/B_{0},\;\tilde{v}=v\sqrt{4\pi \rho_{0}}/B_{0}, \end{array}$$

where *T*_0_ is the duration of the reconnection pulse, *ρ*_0_ is the mass density at the center of the current sheet, *B*_0_ is the magnetic field at the upper boundary, and *L*_0_=*v*_*A*_ *T*_0_.

**Figure 6:**
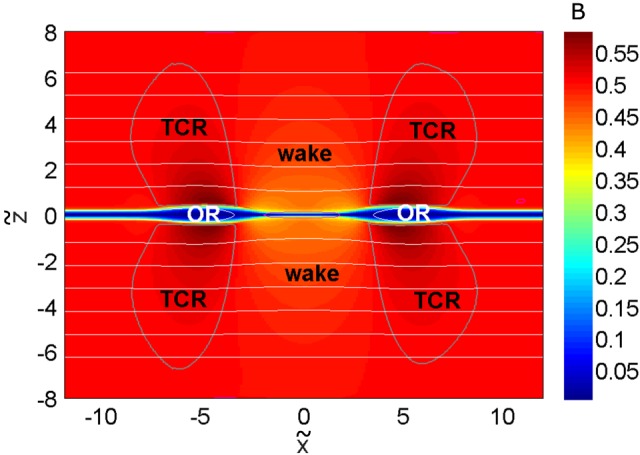
Magnetic field energy in the simulation domain with the three regions under investigation (OR, TCR, and wake) labeled. The boundary of the OR is defined via *v*_*x*_=0, and the boundary between TCR and wake via Δ*W*_*B*_=0. Magnetic field lines are shown in white. $\tilde{x}$, $\tilde{z}$, and *B* are normalized to *v*_*A*_ *T*_0_ and the magnetic field strength at the upper boundary (*B*_0_), respectively.

The boundaries of our calculation domain are assumed to be open. We set the normal derivative zero for each dependent variable at the open boundary. For the simulation a *β* value of 0.3 at the upper and lower boundaries is chosen. Following the approach choosen for the theoretical investigation, we consider three regions (OR, TCR, wake), as displayed in Figure 6.

## 10 Energy Budget—Simulation

**Figure 7:**
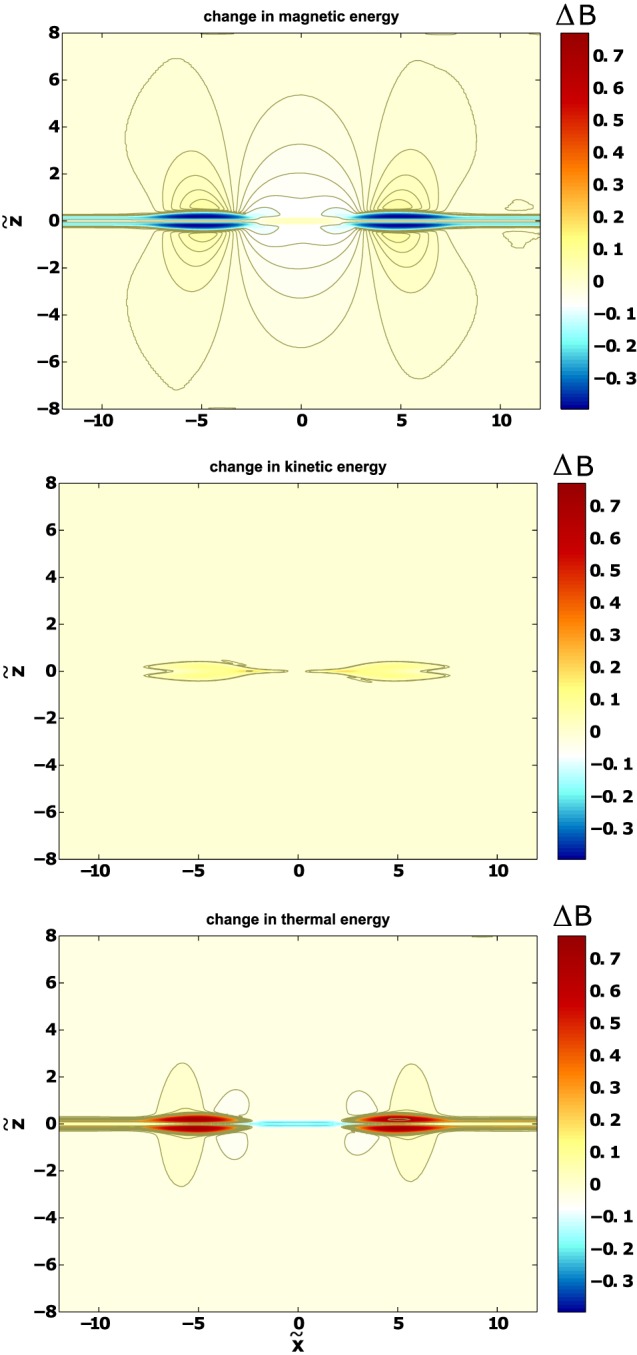
Change in (top) magnetic, (middle) kinetic, and (bottom) thermal energies at time *t* = 9.6598. $\tilde{x}$, $\tilde{z}$, and *B* are normalized to *v*_*A*_ *T*_0_ and the magnetic field strength at the upper boundary (*B*_0_), respectively.

Figure 7 shows the spatial change in all three energy forms (magnetic, kinetic, and thermal), based on the MHD simulation outlined in section 9. As with the analytical model, the same spatial changes in the energies can be observed qualitatively: (a) the magnetic energy decreases in the OR and wake and increases inside the TCR, (b) the acceleration of plasma leads to an increase in the kinetic energy inside the OR, and (c) the thermal energy increases in the OR and TCR and decreases inside the wake. The decrease in thermal energy inside the wake region is primarily due to thermal energy decrease in the preexisting plasma sheet. With this, reconnection leaves behind a cooled plasma sheet, possibly changing the conditions for potential subsequent reconnection.

Changes inside the OR are confined to a smaller region compared to the surrounding area. Consequently, the changes appear qualitatively most significantly inside the OR. However, for a full treatment of the energy changes, it is necessary to integrate over each corresponding area, which is done in the following section.

## 11 Shape of the Outflow Region

**Figure 8:**
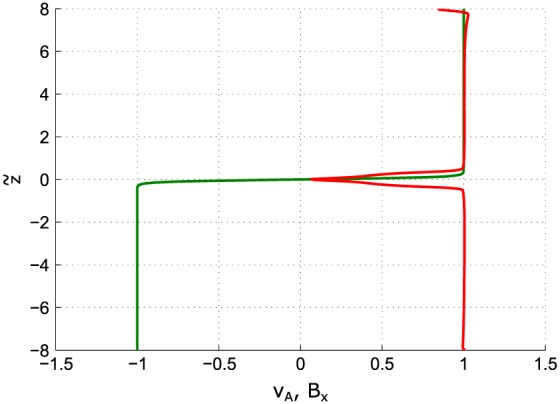
Alfvén speed *v*_*A*_ (red) and magnetic field *B*_*x*_ (green) profiles along vertical direction $\tilde{z}$ at *t* = 0.01. $\tilde{z}$ and *B*_*x*_ are normalized to *v*_*A*_ *T*_0_ and the magnetic field strength at the upper boundary (*B*_0_), respectively.

**Figure 9:**
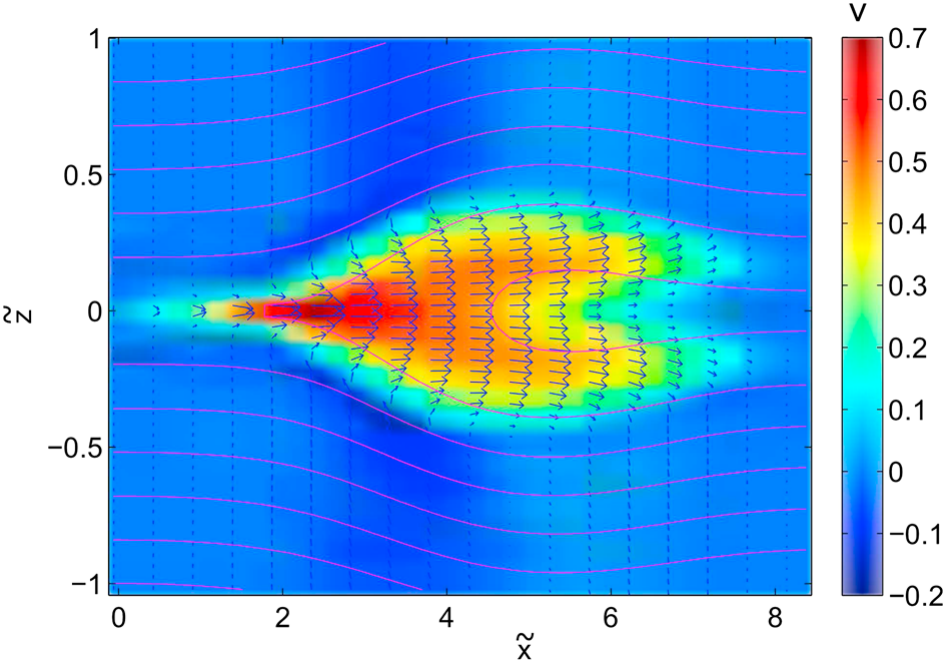
Velocity field of the OR in the simulation at *t* = 10. Magenta lines denote magnetic field lines. $\tilde{x}$, $\tilde{z}$, and *v* are normalized to *v*_*A*_ *T*_0_, and *v*_*A*_, respectively.

**Figure 10:**
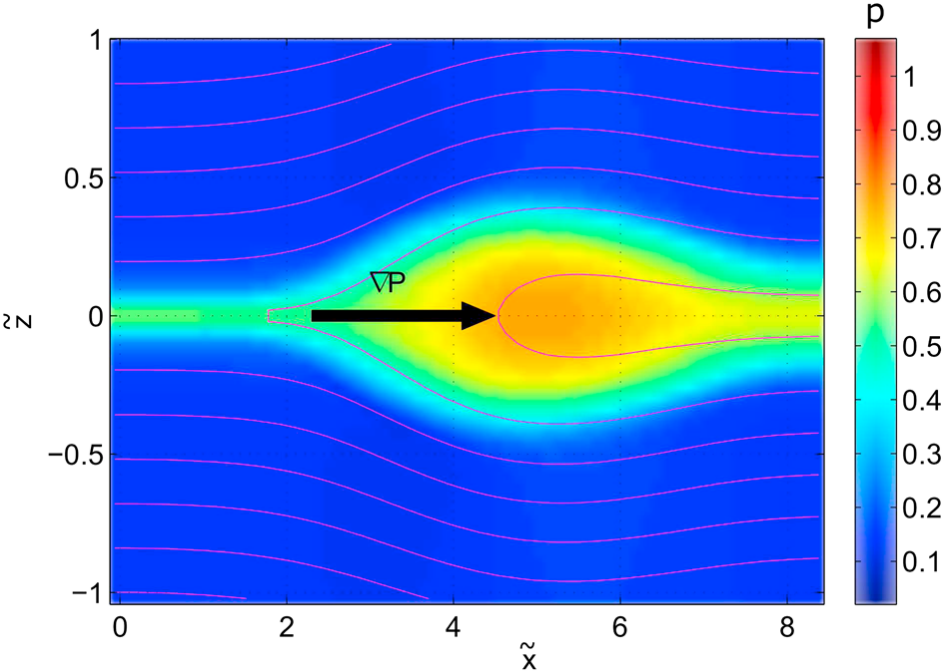
Pressure color contour of the OR at *t* = 10. Magenta lines denote magnetic field lines. $\tilde{x}$, $\tilde{z}$, and *p* are normalized to *v*_*A*_ *T*_0_, and $\frac{B_{0}^{2}}{4\;\pi }$, respectively.

In the analytical model described in section 2 the outflow region can be described via equation (9). With this, the OR exhibits a teardrop‐type shape, as displayed in Figure 1. The analytical model assumes an infinitely thin current sheet. However, for a realistic magnetotail configuration, a current sheet with finite thickness must be considered. The numerical model described in section 9 allows such a consideration. As a result of the continuous vertical density and magnetic field gradients across the current sheet (see Figure 8), the outflow regions exhibit a crab‐hand structure [*Abe and Hoshino*, 2001; *Kiehas et al.*, 2007; *Zenitani et al.*, 2010; *Ugai et al.*, 2011], as shown in Figure 9. The field line nearest to the center of the current sheet is associated with the lowest Alfvén speed. Since the reconnection outflow speed is associated with the Alfvén speed in the inflow region, the speed of initially accelerated plasma is smaller than that of subsequently accelerated plasma, which flows into the reconnection region with field lines associated with higher Alfvén speeds. This leads to slow‐moving plasma in the leading part of the OR and faster‐moving plasma in its trailing part. Additionally, due to the plasma density gradient along *z*, accelerated plasma runs into denser plasma ahead of it and gets diverted around it. Consequently, a crab‐hand‐shaped OR is formed, contrary to the teardrop‐shaped OR in the theoretical model without density gradient in *z*. The higher density in the leading part of the OR also results in the establishment of a pressure gradient pointing into the outflow direction (see Figure 10). This pressure gradient is absent in the theoretical model and vitally important for the understanding of energy conversion.

## 12 Differences Between Theory and Simulation

**Figure 11:**
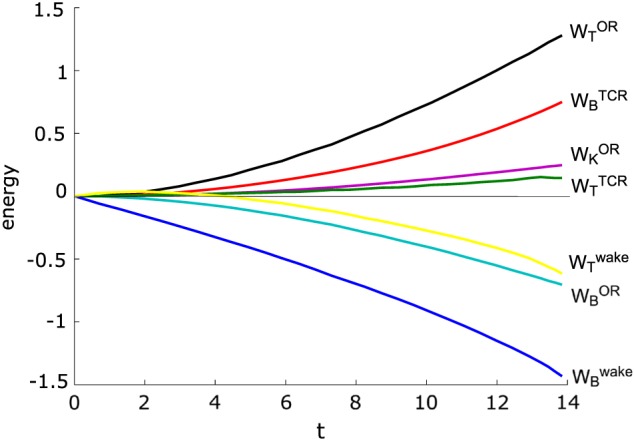
Temporal evolution of each energy form (kinetic, thermal, and magnetic) in the three different regions (OR, wake, and TCR) in the simulation. Time and energies are normalized to *T*_0_ and $\frac{{B_{0}}^{2}}{4\;\pi }\;{\left(v_{A}\;T_{0}\right)}^{2}$, respectively.

Figures 5 and 11 show the temporal evolution of each energy form from the theoretical model and the numerical simulation, respectively. Theory and simulation yield similar results except for the kinetic and thermal energy inside the OR. While the kinetic energy dominates over the thermal energy inside the OR in the theoretical model, the situation is vice versa in the simulation. Previous simulations [*Birn et al.*, 2012] show the same dominance of thermal energy over kinetic energy inside the OR which is also confirmed by observations [*Eastwood et al.*, 2013].

The reason for this discrepancy can be found in the simplifications of the theoretical model, where the current sheet is modeled infinitely thin. Without a gradual magnetic field and plasma density variation over the current sheet, the Alfvén velocity is constant, leading to the same outflow velocity throughout the reconnection process. Furthermore, there is no diversion of outflowing plasma due to the homogeneous plasma density distribution and the absence of a plasma sheet in the theoretical model. Consequently, the OR exhibits a teardrop rather than a crab‐hand shape in the theoretical model and lacks a pressure gradient inside.

## 13 Role of ∇*p*

The thermal and kinetic energy fluxes can be written [*Birn and Hesse*, 2005] as

(51) $$\frac{\mathrm{\partial u}}{\mathrm{\partial t}}=-\nabla \cdot \left[(u+p)v\right]+\eta \;j^{2}+v\cdot \nabla p,$$

(52) $$\frac{\partial }{\mathrm{\partial t}}\;\frac{\rho }{2}\;v^{2}=-\nabla \left(\frac{\rho }{2}\;v^{2}\;v\right)+\frac{v}{c}\cdot \left(j\times B\right)-v\cdot \nabla p,$$

where *u* denotes the internal energy. In the theoretical model the terms **v**·∇*p* on the right‐hand side of equations (51) and (52) are zero. This circumstance reflects the absence of any ∇*p* due to the assumption of an infinitely thin current sheet. However, it is this ∇*p* that increases the thermal energy (equation (51)) and decreases the kinetic energy (equation (52)) for the same amount via the expression **v**·∇*p*. Hence, the pressure gradient, which is present inside the OR under considerations of a finite current sheet, leads to the conversion of kinetic into thermal energy. This circumstance is given in the numerical simulation and explains why the thermal (kinetic) energy in the simulation is larger (smaller) than in the model. As can be seen from comparing Figures 5 and 11, the thermal energy dominates over the kinetic energy inside the OR for an initial configuration with a current sheet of finite thickness (as in the numerical simulation), while the situation is vice versa for an initial configuration with an infinitely thin current sheet (as in the theoretical model). Physically, the dominance of thermal over kinetic energy can be understood by a braking of the flow due to the pressure gradient and the accompanied conversion of kinetic flow energy to thermal energy by compressional heating, as it was pointed out in *Birn et al.* [2010].

## 14 Quasi‐Static Equilibrium

**Figure 12:**
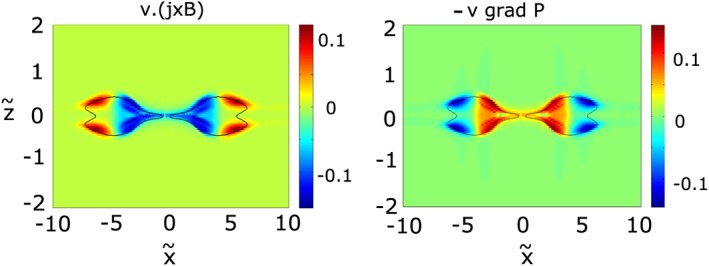
(left) Ampere‐force ( $\frac{v}{c}\cdot (j\times B)$) and (right) pressure gradient (**v**·∇*p*) related terms from equation (52). The black contour shows the edge of the OR. $\tilde{x}$, $\tilde{z}$ are normalized to *v*_*A*_ *T*_0_, and $\frac{v}{c}\cdot (j\times B)$ and **v**·∇*p* to $\frac{{B_{0}}^{2}}{4\;\pi \;T_{0}}$.

**Figure 13:**
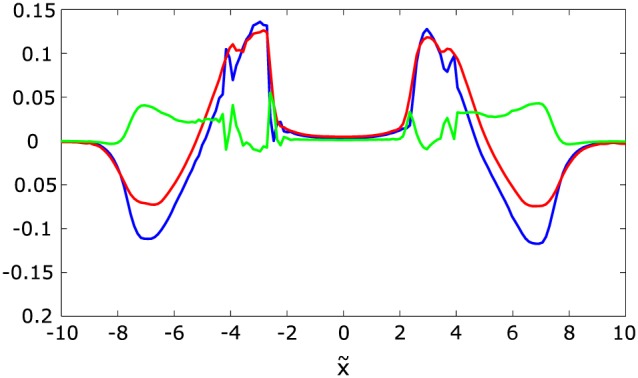
Profiles at $\tilde{z}=0$ along $\tilde{x}$ of ( $\frac{v}{c}\cdot (j\times B)$) (blue) and (**v**·∇*p*) (red) and the difference between both (green). $\tilde{x}$, $\frac{v}{c}\cdot (j\times B)$ and **v**·∇*p* are normalized to *v*_*A*_ *T*_0_ and $\frac{{B_{0}}^{2}}{4\;\pi \;T_{0}}$, respectively.

Using a Sweet‐Parker approach, *Birn and Hesse* [2010] and *Birn et al.* [2010] showed that the Ampere and pressure gradient forces, related to the second and third terms in equation (52), respectively, nearly balance each other. They noted that this result is related to the simulation setup of symmetric boundaries. A quick, qualitative assessment using our open boundary simulation indeed yields differences. Figure 12 shows contour plots of **v**·∇*p* and $\left(\frac{v}{c}\cdot (j\times B)\right)$. Both terms show a similar spatial distribution. Figure 13 displays profiles along *x* as a spacecraft crossing through the ORs at *z* = 0 would measure the value of each term. The difference between the two terms is shown in green. As can be seen the ∇*p* force balances the **j** × **B** force for at least 60%. We see this limited achievement of a force‐free balance due to the open boundaries in our simulation. While a closed magnetic field line configuration might yield full force balance, open boundary conditions provide only partial force balance. However, compared to the analytical model, the appearance of ∇*p*, which is absent in the theoretical model, brings the OR closer to an equilibrium state, which is assumed for the reconstruction of the 2‐D magnetic field configuration [e.g., *Hau and Sonnerup*, 1999; *Hu and Sonnerup*, 2002]. With a stronger pressure gradient, acting against the Ampere force, more kinetic energy gets converted to thermal energy via compressional heating (cf. section 13 and *Birn et al.* [2012]). Under full force balance this process is optimized.

## 15 Summary and Conclusions

**Figure 14:**
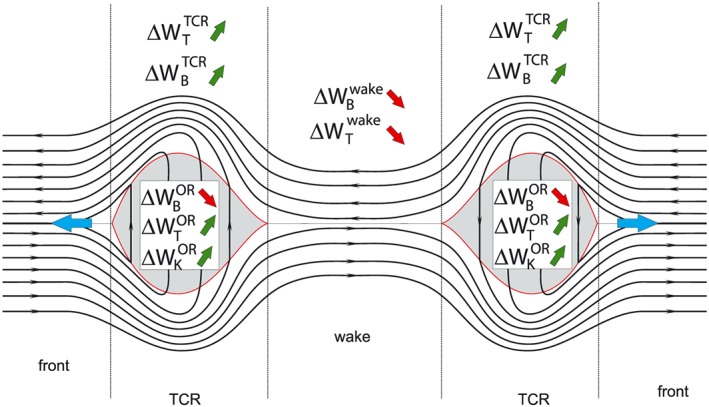
Schematic depiction of the change of thermal, magnetic, and kinetic energies due to reconnection in the three evaluated regions (wake, TCR, and OR).

We evaluated the energy redistribution during time‐dependent magnetic reconnection. For incompressible conditions we find an exact balance between the decrease in magnetic energy and increase in kinetic energy inside the OR, based on the annihilation of magnetic energy in the expanding OR, which corresponds to a magnetic energy flux into the OR, and its conversion to kinetic energy. Hence, all energy needed for the acceleration of plasma is provided by a direct conversion of magnetic energy into kinetic plasma energy. For compressible conditions, the decrease in magnetic energy inside the OR is not sufficient to feed the increase in thermal and kinetic energies inside the OR. This imbalance can be solved by considering also the energy changes in the IR. Due to the depletion of magnetic flux in the wake, the magnetic energy inside the wake decreases. Inside the TCR the magnetic energy increases due to the compression of field lines above and below the OR. In the incompressible case, the increase in magnetic energy inside the TCR is compensated by the decrease of magnetic energy inside the wake. For compressible situations, the decrease in magnetic energy in the wake is insufficient to explain the increase in magnetic energy inside the TCR. By taking into account the decrease of thermal energy in the wake, as well as the increase of thermal energy in the TCR, the energy budget is fully balanced. All together, the increase in thermal and kinetic energies inside the OR is compensated by the decrease of magnetic energy inside the OR and the change in the thermal and magnetic energies in the IR. A summary of the energy changes in each region is shown in Figure 14.

While reconnection heats and accelerates plasma, it leaves plasma in the wake of the OR with a decrease in the thermal energy and hence with less temperature. In that sense reconnection also acts as a refrigerator, as it removes thermal energy from the initial reconnection site and transports it into open space via the OR. This state of the wake plasma might be important for conditions of possible secondary reconnection pulses, as the left behind plasma sheet is now cooler than it was during the first reconnection pulse.

It must be noted that the energy conversion takes place at the shock fronts. Consequently, even after reconnection stopped with the breakdown of the reconnection electric field, energy conversion continues at the moving fronts.

One can define a reconnection efficiency as ratio between released nonthermal (i.e., kinetic) energy and dissipated magnetic energy $\eta_{\mathrm{ef}\;f}=\frac{\Delta W_{k}^{\mathrm{OR}}}{\Delta W_{B}^{\mathrm{wake}}}$ and find as an upper limit *η*_eff_ = 1/2 for both, compressible and incompressible cases. Hence, only half of the available magnetic energy can be converted into kinetic energy. This value corresponds to the efficiency also found for symmetric [*Priest and Forbes*, 2000] and asymmetric [*Birn et al.*, 2010] Sweet‐Parker reconnection.

The analytical model assumes an infinitely thin current sheet, which results in teardrop‐shaped ORs and a homogeneous outflow velocity. As a result of this initial condition in the analytical model, the kinetic energy inside the OR dominates over the thermal energy, which contradicts observations and previous simulation results. Utilizing a numerical simulation, the plasma pressure gradient can be identified as an important player for the conversion of kinetic into thermal energy. Under realistic conditions of a current sheet with finite thickness, the Alfvén velocity is distributed inhomogeneously across the current sheet, leading to an inhomogeneously distributed outflow speed which is smaller at the leading front of the OR than at the edges or the trailing part of it. Furthermore, less dense plasma gets accelerated into denser plasma at the front of the OR. Consequently, a pressure gradient builds up inside the OR. This pressure gradient enters the equations for the thermal and kinetic energies, reducing the kinetic energy for **v**·∇*p* and enhancing the thermal energy for the same amount. Physically, this means that the plasma has to work against the pressure gradient, leading to a conversion of kinetic into thermal energy via compressional heating. Furthermore, the gas pressure partially balances the Ampere force, bringing the OR closer to a quasi‐steady equilibrium of the OR, which is generally assumed for 2‐D reconstruction techniques of the magnetic field line configuration.

## Appendix A Overview of Equations

### A.1

Outflow region:

(A1) $$W_{k}^{\mathrm{OR},Q1}=\frac{v_{A}\;B_{0}}{8\pi }\;G(t).$$

(A2) $$\Delta W_{B}^{\mathrm{OR},Q1}=-\frac{v_{A}\;B_{0}}{8\pi }\frac{\rho_{0}}{\tilde{\rho }}\;G(t).$$

(A3) $$\Delta W_{T}^{\mathrm{OR},Q1}=\frac{v_{A}\;B_{0}}{8\;\pi }\frac{\rho_{0}}{\tilde{\rho }}\frac{1}{\gamma -1}G(t).$$

Inflow region:

(A4) $$\Delta W_{B}^{\mathrm{IR},Q1}(x=v_{A}\;t)=-\frac{v_{A}\;B_{0}}{4\pi }\left(\frac{\rho_{0}}{\tilde{\rho }}\right)\;G(t).$$

(A5) $$\Delta W_{T}^{\mathrm{IR},Q1}(x=v_{A}\;t)=-\frac{\gamma \;\beta }{\gamma \;-1}\frac{v_{A}\;B_{0}}{8\;\pi }\left(1-\frac{\rho_{0}}{\tilde{\rho }}\right)\;G(t).$$

Wake:

(A6) $$\Delta W_{B}^{\mathrm{wake},Q1}=-\frac{v_{A}\;B_{0}}{4\pi }\;t\;F_{0},$$

(A7) $$\Delta W_{T}^{\mathrm{wake},Q1}=-\frac{\gamma \;\beta }{\gamma \;-1}\frac{v_{A}\;B_{0}}{8\pi }\;t\;F_{0},$$

TCR:

(A8) $$\Delta W_{B}^{\mathrm{TCR},Q1}=\frac{v_{A}\;B_{0}}{4\pi }\left(\frac{\rho_{0}}{\tilde{\rho }}\right)\;t\;F_{0},$$

(A9) $$\Delta W_{T}^{\mathrm{TCR},Q1}=\frac{\gamma \;\beta }{\gamma \;-1}\frac{\rho_{0}}{\tilde{\rho }}\frac{v_{A}\;B_{0}}{8\pi }\;t\;F_{0}.$$

## Appendix B Displacement Vector

### B.1

We can define a displacement vector as

(B1) $$v=\frac{\partial \xi }{\mathrm{\partial t}}.$$

Inserting this in the equation for continuity,

$$\frac{\partial \rho }{\mathrm{\partial t}}+\nabla \cdot (\rho \;v)=0,$$

we find

(B2) $$\rho^{\left(1\right)}=-\rho_{0}\nabla \xi .$$

Inserting equation (B1) into Faraday's law,

(B3) $$\frac{\partial B}{\mathrm{\partial t}}=\nabla \times \left(v\times B\right),$$

we find for first‐order disturbances in *B*_*z*_,

(B4) $$B_{z}^{\left(1\right)}=B_{0}\;\frac{\partial }{\mathrm{\partial x}}\;\xi_{z},$$

and hence, the *z* component of the displacement vector can be found as

(B5) $$\xi_{z}=\frac{1}{B_{0}}\int {B_{z}}^{\left(1\right)}dx.$$
